# Morphological Priming Effects in L2 English Verbs for Japanese-English Bilinguals

**DOI:** 10.3389/fpsyg.2022.742965

**Published:** 2022-07-28

**Authors:** Jessie Wanner-Kawahara, Masahiro Yoshihara, Stephen J. Lupker, Rinus G. Verdonschot, Mariko Nakayama

**Affiliations:** ^1^International Graduate Program in Language Sciences, Graduate School of International Cultural Studies, Tohoku University, Sendai, Japan; ^2^Research Institute for Letters, Arts, and Sciences, Waseda University, Tokyo, Japan; ^3^Department of Psychology, University of Western Ontario, London, ON, Canada; ^4^Max Plank Institute for Psycholinguistics, Nijmegen, Netherlands

**Keywords:** morphological priming, fuzzy lexicon, bilinguals, L2 English, proficiency

## Abstract

For native (L1) English readers, masked presentations of past-tense verb primes (e.g., fell and looked) produce faster lexical decision latencies to their present-tense targets (e.g., FALL and LOOK) than orthographically related (e.g., fill and loose) or unrelated (e.g., master and bank) primes. This facilitation observed with morphologically related prime-target pairs (morphological priming) is generally taken as evidence for strong connections based on morphological relationships in the L1 lexicon. It is unclear, however, if similar, morphologically based, connections develop in non-native (L2) lexicons. Several earlier studies with L2 English readers have reported mixed results. The present experiments examine whether past-tense verb primes (both regular and irregular verbs) significantly facilitate target lexical decisions for Japanese-English bilinguals beyond any facilitation provided by prime-target orthographic similarity. Overall, past-tense verb primes facilitated lexical decisions to their present-tense targets relative to both orthographically related and unrelated primes. Replicating previous masked priming experiments with L2 readers, orthographically related primes also facilitated target recognition relative to unrelated primes, confirming that orthographic similarity facilitates L2 target recognition. The additional facilitation from past-tense verb primes beyond that provided by orthographic primes suggests that, in the L2 English lexicon, connections based on morphological relationships develop in a way that is similar to how they develop in the L1 English lexicon even though the connections and processing of lower level, lexical/orthographic information may differ. Further analyses involving L2 proficiency revealed that as L2 proficiency increased, orthographic facilitation was reduced, indicating that there is a decrease in the fuzziness in orthographic representations in the L2 lexicon with increased proficiency.

## Introduction

Word recognition studies involving bilinguals have focused mainly on understanding the relationship between first (L1) and second language (L2) representations. Some of this focus stems from the debate on language selectiveness vs. non-selectiveness of lexical access (see Jiang, 2015 for a review), which now seems to favor the language non-selective access hypothesis (see Dijkstra, 2005 for a review). Understanding the structure and inner workings of the bilingual’s L2 lexicon itself has now become another important focus of bilingual visual word recognition studies (e.g., Bordag et al., 2021). Relevant studies commonly address the question of whether they are the same as or different from those of the L1 lexicon. Some studies have suggested that L2 representations are fuzzy, meaning that lexical items in a second language may be encoded in a less precise manner than in the first language of a bilingual (Cook et al., 2016). This idea has been investigated in some detail for form-meaning mappings (in the L2). In contrast, there is, at this point, little information concerning whether the same is true for morphological level representations.

With respect to visual word recognition, some studies have reported that proficient L2 readers produce the same pattern of results as L1 readers (e.g., Witzel et al., 2011, transposed letter/character priming effects; Nakayama et al., 2013, frequency attenuation of repetition priming effects), suggesting that certain aspects of how L2 readers process and represent L2 words seem similar to those of L1 readers. Other studies with proficient L2 readers, however, have shown different patterns of results from those of L1 readers. For example, the word frequency effect has been found to be greater in L2 than in L1 (e.g., Duyck et al., 2008). It has also been shown that near-homophones (ROCK vs. LOCK) can produce an interference effect in a semantic relatedness judgment task (i.e., are ROCK and KEY related?) in L2 but not in L1 readers (Ota et al., 2009). This latter result seems to indicate that certain phono-lexical representations (e.g., those having a non-native /l/ - /r/ contrast) might indeed be fuzzy (i.e., stored imprecisely) and are therefore hard to separate for Japanese-English bilinguals. Furthermore, lexical competition, the process during which orthographically similar words compete with each other during the word recognition process (e.g., Segui and Grainger, 1990; Davis and Lupker, 2006), appears to be absent, or at least greatly diminished, for L2 readers (e.g., Qiao and Forster, 2017; Nakayama and Lupker, 2018; Jiang, 2021). Weak lateral inhibition in L2 learners has also been reported in the auditory domain (Gor and Cook, 2020). Although different behavioral results do not inevitably indicate that L1 and L2 lexicons are organized qualitatively differently (e.g., see Brysbaert et al., 2017), it is certainly the case that understanding both the similarities and differences between how words are processed and represented in the L1 and L2 lexicons is critical to gaining a clear picture of the bilingual language system.

In the present research, we explored a potential difference between L2 and L1 English lexicons by examining the representations of morphological relationships of L2 words for Japanese-English bilinguals. As previous studies have shown that representations for L2 word forms appear to differ from those for L1 words (e.g., Qiao and Forster, 2017; Nakayama and Lupker, 2018),^1^ the question of whether differences between L1 and L2 representations also exist for representations of morphological relationships is clearly of interest. The type of morphological relationship examined here was that between past and present tense verb forms. Because of the extensiveness of the literature on this issue, we limit our discussion to masked priming visual word recognition experiments that investigate L2 English processing.

We focused on this particular morphological relationship because previous studies examining L1 (native) English readers have reliably observed significant masked priming effects between past-tense verb primes and their present-tense targets (fell-FALL, boiled-BOIL). Such priming effects indicate that there are special connections between the two types of words, due to their morphological relationship in the L1 English reader’s lexicon (e.g., Forster et al., 1987; Crepaldi et al., 2010). In contrast to L1 studies, previous studies investigating L2 English readers have reported mixed results: some studies reporting that L2 readers produce priming patterns similar to those of L1 readers (Feldman et al., 2010; Voga et al., 2014) and others reporting that they do not (e.g., Silva and Clahsen, 2008; Clahsen et al., 2013). The latter pattern suggests that non-native readers of a language are not as sensitive to the morphological structure, or to morphological exceptions, as native readers are. Therefore, the L2 morphological makeup of particular words may perhaps constitute another source of fuzziness in the mental lexicon (similar to meaning-form mappings; Cook and Gor, 2015). However, we should point out that the question of whether L2 readers represent the relationship of past-tense verbs and their present-tense forms similarly to L1 readers does not have a clear answer at present.

In these masked priming lexical decision experiments, researchers typically compare the speed at which targets are responded to when the targets are preceded by a brief (40–60 ms) presentation of a prime that is in some way related to its target (e.g., orthographically, phonologically, semantically, morphologically, etc.) versus a prime that is unrelated to its target. When responses to targets are differentially affected by related versus unrelated primes (i.e., faster or slower), the latency difference is called a priming effect. This priming effect is thought to occur due to primes that are in some way related to their targets pre-activating their target’s representations based on that relatedness. Significant priming effects, therefore, indicate that representations of primes and targets share processing structures as a result of their related property. Priming based on morphological relationships, that is, a morphological priming effect, is, therefore, thought to reflect some kind of connectedness between the representations of prime and target words based on morphology.

Forster et al. (1987) were among the first to demonstrate masked priming effects of the sort being examined here, that is, between irregular inflectional past-tense verbs and their present-tense forms (e.g., kept-KEEP) for L1 English readers. In their monolingual experiment using a 60 ms prime duration, targets primed by their past-tense forms were responded to significantly faster than the same targets primed by unrelated primes (e.g., kept-KEEP vs. navy-KEEP). Past-tense primes, in fact, facilitated target recognition as much as identity primes did (e.g., keep-KEEP = kept-KEEP < navy-KEEP; 36 ms vs. 37 ms effects), indicating that a past-tense verb has the ability to access or pre-activate present-tense verb representations as efficiently as the verb itself for native speakers. Using regular past-tense and present-tense form pairs (e.g., boiled-BOIL), Silva and Clahsen (2008) also found significant priming effects relative to unrelated primes (jump-BOIL—a 55 ms effect). Similar to Forster et al. (1987), the size of the morphological priming effect was not statistically different from the size of the parallel identity priming effect (67 ms). Crepaldi et al. (2010), using a slightly shorter prime duration (42 ms), replicated the significant priming effect for irregular past-tense and present-tense verb pairs (e.g., fell-FALL vs. hope-FALL, 25 ms) with stringently controlled stimuli. Importantly, they added a crucial control condition, orthographic primes (e.g., fill-FALL). Specifically, the orthographic similarity of their orthographic primes and targets was matched to the orthographic similarity of their morphological primes and targets. The inclusion of orthographic primes thereby guarded against the possibility that any priming observed for the fell-FALL pairs might have been orthographically based. The morphological primes produced a significant (21 ms) priming effect using the orthographic condition as a baseline.

Finally, Pastizzo and Feldman (2002) tested both regular and irregular verbs in a single experiment and found significant morphological priming effects, again using orthographic control primes as baselines, for both irregular (e.g., fell-FALL vs. fill-FALL; a 33 ms effect) and regular verbs (billed-BILL vs. billion-BILL; a 44 ms effect), although only a non-significant (15 ms) effect was observed for a different group of irregular verb pairs, that is, pairs that had low form overlap and varied in word length (taught-TEACH vs. taunts-TEACH). As such, for L1 English readers, priming effects for past-tense and present-tense verb pairs have been reliably observed, and such is the case for both regular (Pastizzo and Feldman, 2002; Silva and Clahsen, 2008; Feldman et al., 2010) and irregular verb pairs (Forster et al., 1987; Crepaldi et al., 2010; Feldman et al., 2010). Reliable priming effects observed for past-tense and present-tense word pairs by L1 English readers have been taken to imply that representations of the two words are shared and/or intimately connected in L1 lexicons due to their morphological relationship.

What should be noted at this point is that there is some disagreement among morphological processing models as to how the representations of past- and present-tense forms are shared and/or connected in the L1 lexicon. One point of contention is whether past-tense forms are represented and, hence, processed differently based on their inflectional regularity or not. Some models have proposed that two different cognitive mechanisms are employed for processing past-tense forms (see Pinker and Ullman, 2002). In these models, regular past-tense forms are decomposed *via* the application of morpho-syntactic rules (i.e., verb stem + regular past-tense suffix). Only the verb stem’s representation is stored in the lexicon, which is identical to the representation of the present-tense form. For this reason, and consistent with Silva and Clahsen’s (2008) results, regular past-tense forms would be expected to prime their present-tense forms just as efficiently as the present-tense forms themselves in masked priming experiments. In contrast, irregular past-tense forms cannot be decomposed by morpho-syntactic rules. Therefore, they are stored in their full form in the lexicon. Morphological priming for irregular past- and present-tense forms is thus a result of the two forms being connected by their formal and morphological/semantic relationship in the lexicon. In contrast to this view of morphological processing, other models require only one mechanism. Some of these models posit explicit representations relating to the morphological structure of words in the lexicon, while others do not (for a review, see Feldman and Weber, 2012; Milin et al., 2018). Despite differences among the models, past-tense forms are assumed to be processed similarly regardless of their inflectional regularity, and morphological priming is explained by shared and/or connected lexical representations for past- and present-tense forms.

In contrast to the consistent results for L1 readers, previous studies produced inconsistent results for L2 readers (in line with the idea that L2 readers might have inexact, or fuzzy, morphological representations). Some experiments showed no morphological priming effect for past-tense verb primes and their present-tense targets in situations, in which an effect has been observed for L1 English readers (e.g., Silva and Clahsen, 2008; Clahsen et al., 2013). Silva and Clahsen (2008), for instance, had both Chinese-English and German-English bilinguals make lexical decisions to regular verb targets (e.g., BOIL) that were preceded by past-tense primes (e.g., boiled), identity primes (e.g., boil), or unrelated primes (e.g., jump). Although their control group of L1 readers showed a significant priming effect for morphologically related pairs (55 ms), which was statistically as strong as the identity priming effect (67 ms), for the two groups of L2 readers, facilitation from past-tense verb primes was absent. Despite the null morphological priming effect, both L2 groups nevertheless showed significant identity priming effects (84 ms and 59 ms for Chinese- and German-English bilinguals, respectively), indicating that those individuals were capable of processing masked L2 primes. The lack of a morphological priming effect for L2 English readers was replicated by Clahsen et al. (2013), in which the same stimulus set used by Silva and Clahsen (2008) was tested with a group of Arabic-English bilinguals. Past-tense primes again failed to facilitate target recognition for bilinguals relative to unrelated primes (e.g., boiled-BOIL = jump-BOIL), although a significant repetition priming effect was again observed for the L2 readers (e.g., boil-BOIL < jump-BOIL). Lack of priming effects from past-tense verb primes in these experiments suggests that at least for regular verbs, there is no underlying connection or shared representation between past-tense verbs and their present-tense forms in the lexicons of L2 readers. In line with the view that past-tense forms are processed differently depending on their regularity, Silva and Clahsen (2008) and Clahsen et al. (2013) have taken their results to suggest that morpho-syntactic processing is less effective for L2 readers than for L1 readers.

Other studies using past- and present-tense pairs with L2 English readers, on the other hand, did find a significant pattern of priming effects that was similar to the one typically observed for L1 English readers (Feldman et al., 2010; Voga et al., 2014), suggesting that the representations and processing of past-tense forms for L2 and L1 readers could be similar. Voga et al. (2014), for example, using the same set of stimuli used by Silva and Clahsen (2008), but with a slightly shorter prime duration (50 ms), found significant priming effects from regular past-tense primes for their Greek-English bilinguals (e.g., boiled-BOIL < jump-BOIL). Furthermore, the past-tense primes facilitated their targets to a degree that was statistically equivalent to that of the identity primes (66 ms and 54 ms effects, respectively). This priming pattern is exactly the priming pattern observed with L1 English readers by Silva and Clahsen (2008).

Some additional support for similar underlying connections among morphologically related word pairs for L1 and L2 English readers (i.e., past-tense inflectional morphological pairs) comes from a study by Feldman et al. (2010), a study that is directly relevant to the present experiments. Feldman et al. tested a group of Serbian-English bilinguals with the same set of regular verb pairs and the two types of irregular verb pairs (e.g., billed-BILL, fell-FALL, taught-TEACH) used with L1 readers by Pastizzo and Feldman (2002). The data from the bilinguals showed that, relative to *orthographic control primes* (billion-BILL), a significant 23 ms priming effect was observed for regular verbs (billed-BILL) although not for the irregular verbs irrespective of the degree of form overlap (fell-FALL = fill-FALL, a 3 ms difference, taught-TEACH = taunts-TEACH, an 11 ms difference). A re-analysis of Pastizzo and Feldman’s (2002) L1 data (i.e., 9 items that produced high error rates for Serbian-English bilinguals were removed for a better and more direct comparison of the L1 vs. L2 data), however, did show reliable morphological priming effects for irregular verb types (20 and 19 ms effects), although the effect for regular verbs (42 ms) was somewhat larger.

What is also important to note is that when Feldman et al. (2010) examined priming effects measured against *unrelated primes*, a somewhat different pattern emerged. Specifically, for the L2 readers, past-tense primes produced significant priming effects that were not statistically different across the three verb types (23, 33, and 32 ms effects for fell-FALL, taught-TEACH, and billed-BILL type pairs, respectively). The same priming pattern was also observed for L1 readers; past-tense primes produced significantly faster responses to targets, and there was no interaction with verb type (20, 22, and 30 ms effects). Therefore, in general, Feldman et al.’s results indicate that L2 readers produce a similar result pattern to that of L1 readers. Further, their results suggest that any behavioral difference between L1 and L2 readers in these types of experiments may be due to the differential impact of orthographic primes on the word recognition process.

More specifically, for L2 readers, orthographic similarity almost always facilitates target processing (Nakayama and Lupker, 2018; Jiang, 2021; Kida et al., 2022; also see Qiao and Forster, 2017). For example, Nakayama and Lupker (2018), using a 67 ms prime duration, reported that orthographically related pairs such as time-TILE produced facilitation for Japanese-English bilinguals, even though the same prime-target pairs produced an inhibitory effect for L1 English readers. For L1 readers, the inhibitory effect from orthographically similar primes is assumed to occur through the process of lexical competition among the representations activated by the prime. That is, due to the precision of prime encoding, the prime’s lexical representation successfully competes with and inhibits the target’s representation (as well as all other activated representations). This competition/inhibition process delays the target’s lexical representation from reaching the recognition threshold when it is presented for a lexical decision. In contrast, for L2 readers, facilitation from orthographic relationships can be explained as a consequence of fuzziness, that is, that L2 word forms may be encoded in a less precise manner than L1 forms (e.g., Cook and Gor, 2015). Essentially, for L2 readers, representations of words with similar forms are not easily distinguishable. As a result, many orthographically similar candidates are activated by the masked prime and, equally importantly, remain active at the point that the target is presented because the prime does not prevail in the competition process (see Footnote 1). The result is that orthographically similar prime-target pairs virtually always facilitate L2 word recognition. Consequently, when examining morphological relationships in the L2 lexicon, it is critical that the impact of facilitation due to orthographic similarity be controlled because morphologically related word pairs are typically orthographically similar. That is, for L2 readers, if morphological priming effects are measured by using unrelated primes as the baseline, those effects would be contaminated by the effects of orthographic similarity.

Matching lexical and participant characteristics that could also differentially affect response times between the relevant conditions is also crucial. In studies that examine morphological priming, controlled lexical characteristics typically include the frequency of words, neighborhood density, and the degree of prime-target orthographic overlap (e.g., Crepaldi et al., 2010; Feldman et al., 2010). Further, a general assumption in designing the stimuli for this type of experiment is that these characteristics are similar across all participants. This assumption seems reasonable for L1 readers tested in most studies, who are typically college/university students, as their background in acquiring their L1 is likely to be relatively homogeneous. However, one would expect there to be more variability in such characteristics among L2 readers. Factors such as a participant’s age of acquisition of their L2 (e.g., Veríssimo et al., 2018) and L1 background (e.g., Nakayama and Lupker, 2018) could affect how the participant processes words, or at least which words the participant is familiar with and to what degree (e.g., Brysbaert et al., 2017). Research with L1 readers has also shown that certain language skills may be related to how precisely words are represented in the lexicon (e.g., Andrews and Lo, 2012), and it is possible that this conclusion may hold true for L2 readers as well. At present, however, there appears to be no agreed-upon method for gauging language skills in L2 readers. Researchers typically use any one of a number of different L2 proficiency tests to assess or control the L2 language skills of participants, and these tests tend to evaluate different types of language skills (e.g., vocabulary, grammar, and comprehension) for different types of settings (e.g., daily communication, business, and academia). It is, of course, far from clear as to whether the scores from these different tests are reflecting L2 proficiencies in a similar way, making it somewhat difficult to compare the results from morphological priming studies with L2 readers.

Essentially, previous studies focused on the morphological relationship of past-tense verbs and their present-tense forms with L2 readers have not yielded fully consistent results. In the present research, we conducted two masked priming lexical decision experiments with Japanese-English bilinguals in order to provide additional empirical evidence concerning the potential development of a special underlying connection in those readers’ lexicons as a result of the morphological relationship between the past- and present-tense forms. Experiment 1 was designed based on Feldman et al.’s (2010) experiment with Serbian-English bilinguals. We followed Feldman et al.’s design because priming effects were assessed relative to both unrelated primes and orthographic control primes in that experiment. Because both bilinguals and L1 readers were tested using the same procedure and stimuli in Feldman et al.’s experiments, we wished to determine how our bilingual results, obtained in a similar experimental setting, would look in reference to their results.^2^

In summary, the purpose of the present research was to investigate whether the representations of morphological relationships found in L1 English readers’ lexicons are similar to those found in L2 English readers’ lexicons. If a pattern paralleling that shown by L1 English readers is found, then morphological representations are likely encoded in a similar fashion in the mental lexicon of L2 English readers. Specifically, we examined the question of whether representations of inflectional verbal morphology are present in the L2 English lexicon of Japanese-English bilinguals by conducting two masked priming experiments. The setup of the stimuli and the experimental design followed largely those used by Feldman et al. (2010), although a new set of stimuli was selected in order to better suit the breadth of English vocabulary knowledge of our particular bilingual groups. As the results of Experiment 1 were not entirely conclusive with respect to our research question, Experiment 2 was conducted to test the replicability of the main results found in Experiment 1.

## Experiment 1

### Method

#### Participants

A total of 93 Japanese-English bilinguals participated in Experiment 1. Forty-five were recruited from Tohoku University and 48 were recruited from Waseda University. Data collection was conducted in each respective institution. The participants’ L1 language was Japanese, and they were reasonably proficient in English (i.e., they all obtained scores equal to or higher than 610 on TOEIC or 530 on TOEFL ITP or Grade 2 on EIKEN; Eiken Foundation of Japan, n.d.).^3^ Fifty-one participants were male and 42 were female. The mean age of participants at the time of the experiment was 20.85 (*SD* = 3.22). The age they started learning English was, on average, 9.81 (*SD* = 3.40). The time they had spent in an English-speaking region was, on average, 6.32 months (*SD* = 19.59). Each participant received a 1,000-yen gift card (roughly equivalent to US$9.00) for their participation.

#### Stimuli

A total of 81 verbs were selected as targets. Following Feldman et al. (2010), there were three types of verb conditions, each involving 27 targets: (1) Regular verbs (REG) were verbs that take the “-ed” ending to form the past tense (e.g., look-looked; dream-dreamed), (2) Irregular Length Preserved verbs (IRLP) were verbs which do not take the “-ed” ending and, therefore, their past tense is formed irregularly; however, their present- and past-tense forms have the same letter length (e.g., fall-fell; sell-sold), and (3) Irregular Length Varied verbs (IRLV) were verbs which do not take the “-ed” ending, their past tense is formed irregularly and their present- and past-tense forms have different letter lengths (e.g., meet-met; pay-paid). Each target verb was paired with three types of primes: a morphological prime that was the past-tense form of its target (e.g., looked-LOOK, fell-FALL, met-MEET), an orthographic prime that was a word that was orthographically similar but was not morphologically or semantically related to its target and was similar in length to the morphological prime (e.g., loose-LOOK, fill-FALL, and men-MEET), an unrelated prime that was a word that was orthographically, morphologically, and semantically unrelated to its target and was exactly the same length as the morphological prime (e.g., master-LOOK, bank-FALL, and lab-MEET). (See Table 1 for information concerning prime and target characteristics.)

**Table 1:** Lexical characteristics and examples of prime-word target pairs used in Experiment 1.

		Prime type			Targets
		MORPH	ORTH	UNREL	
**IRLP**		**fell**	**fill**	**bank**	**FALL**
	Frequency	91 (152.2)	69 (138.3)	95 (143.1)	512 (1131.7)
	Length	4.3 (0.5)	4.3 (0.5)	4.3 (0.5)	4.3 (0.5)
	Neighbors	8.0 (4.9)	7.6 (4.1)	7.0 (5.7)	8.8 (4.5)
	% Overlap	60 (20.9)	56 (18.9)	8 (11.4)	
	LD	0.39 (0.19)	0.44 (0.19)	0.92 (0.12)	
**IRLV**		**paid**	**pair**	**jump**	**PAY**
	Frequency	139 (195.1)	63 (120.8)	104 (147.2)	444 (852.3)
	Length	4.5 (1.1)	4.4 (1.1)	4.5 (1.1)	4.3 (0.9)
	Neighbors	6.7 (4.7)	7.4 (5.3)	6.6 (4.6)	8.6 (5.2)
	% Overlap	54 (27.7)	50 (23.4)	6.0 (10.5)	
	LD	0.54 (0.43)	0.62 (0.41)	1.12 (0.31)	
**REG**		**looked**	**loose**	**master**	**LOOK**
	Frequency	97 (141.5)	40 (73.8)	97 (165.1)	450 (613.6)
	Length	6.2 (0.8)	5.9 (1.0)	6.2 (0.8)	4.2 (0.8)
	Neighbors	4.4 (2.4)	1.9 (2.3)	4.1 (2.5)	7.9 (4.2)
	% Overlap	67 (4.0)	52 (12.1)	5.0 (8.1)	
	LD	0.49 (0.09)	0.68 (0.26)	1.36 (0.15)	

Values in word frequencies (per million words) and the number of neighbors were according to the English Lexicon Project (Balota et al., 2007). LD refers to the Levenshtein Distance (Levenshtein, 1966).

Because we expected that it would be important to calculate our morphological priming effects based on using the orthographic primes as a control, an effort was made to select orthographic and morphological primes that were equally orthographically similar to their targets. As was done by Feldman et al. (2010), the proportion of letters repeated in the same position between the prime and target was used as a measure of orthographic similarity. We calculated this measure by dividing the number of identical characters in the same letter position between primes and targets by the letter length of the prime and then multiplying it by 100. Therefore, a value of 100 means that the prime and target are exactly the same, whereas a value of 0 means that not a single letter is shared in the same position between the prime and its target. In Table 1, we also report the Levenshtein distances (Levenshtein, 1966) between prime-target pairs as an additional reference of orthographic similarity. To take the differential letter lengths into account (i.e., IRLV and REG conditions), we calculated the normalized Levenshtein distance, where the distance between the prime and target was divided by the number of letters in the longer word stimulus. Hence, the value varies from 0 to 1 with smaller values indicating a higher degree of similarity.

REG, IRLP, and IRLV verb targets were matched in their mean word frequencies, word lengths, and numbers of neighbors (Coltheart et al., 1977), all *F*s < 1. For primes, strict matches were difficult to achieve on some lexical characteristics in certain conditions, mainly due to the fact that the number of irregular verbs is relatively small in English. Further, because late-bilinguals would know a smaller number of words in English than L1 English readers, our stimulus selection had to be even more restrictive. An effort was made, however, to match the lexical characteristics of the primes as much as possible.

For the primes paired with REG targets, repeated measure ANOVAs confirmed that the morphological, orthographic, and unrelated primes were matched on their mean word frequencies [*M*s = 97, 40, 97, respectively, *F*(2, 52) = 1.75, *p* > 0.18] and word lengths [*M*s = 6.2., 5.9., and 6.2., *F*(2, 52) = 2.75, *p* > 0.07]. Despite our best efforts, the prime-target orthographic similarities were higher for morphological primes (*M* = 67%, e.g., looked-LOOK) than for orthographic primes [*M* = 52%, e.g., loose-LOOK, *t*(26) = 6.06, *p* < 0.001]. Unrelated primes had significantly lower prime-target orthographic similarity than both morphological and orthographic primes (*M* = 5%, master-LOOK, *p*s < 0.001). Lastly, morphological and unrelated primes had a statistically equivalent number of neighbors [*N*s = 4.4 and 4.1, *t*(26) = 1.0, *p* = 0.33]; however, orthographic primes had a significantly lower number of neighbors (*N* = 1.9, *p*s < 0.001), due to the fact that matching on prime-target orthographic similarity was made a priority over the primes’ neighborhood sizes.

For the primes paired with IRLP targets, morphological, orthographic, and unrelated primes were statistically matched on their mean word frequencies (*Ms* = 91, 69, 95, *F* < 1), word lengths (*M*s = all 4.3), and neighborhood sizes (*Ms* = 8.0, 7.6, 7.0, *F* < 1). The prime-target orthographic similarity was matched between morphological primes (*M* = 60%, e.g., fell-FALL) and orthographic primes (*M* = 56%, e.g., fill-FALL), *t* (26) = 1.28, *p* > 0.21. Unrelated primes had a significantly lower prime-target orthographic similarity than both morphological and orthographic primes (*M* = 8%, e.g., bank-FALL, *p*s *< 0*.001).

Finally, for the primes paired with IRLV targets, morphological, orthographic, and unrelated primes were statistically matched on their mean letter lengths [*M* = 4.5, 4.4, 4.5, *F*(2, 52) = 2.85, *p* > 0.06] and mean numbers of neighbors (*M* = 6.7, 7.4, 6.6, *F* < 1). The prime-target orthographic similarity was matched between morphological primes and their targets (*M* = 54%, e.g., fell-FALL) and orthographic primes and those same targets (*M* = 50%, e.g., fill-FALL, *t* < 1). Unrelated primes had significantly lower prime-target orthographic similarity than both morphological and orthographic primes (*M* = 6%, *p*s *< 0*.001). A variable that we could not statistically match in this condition was the mean word frequencies; morphological primes had statistically higher mean word frequency (*M* = 139) than both orthographic primes (*M* = 63) and unrelated primes (*M* = 104), *p*s < 0.05, which were not statistically different, *t*(26) = −1.55, *p* > 0.10. The fact that the orthographic primes were lower-frequency was not likely problematic, as Nakayama and Lupker (2018) showed that the facilitation effect from orthographically similar primes for Japanese-English bilinguals is not affected by whether they are words or non-words (non-words have a frequency of 0). For word targets, three presentation lists (List A, List B, and List C) were created in such a way that within a list, a third of the word targets were primed by the morphological primes, a third by the orthographic primes, and a third by the unrelated primes. Across the lists, each word target was primed by each of the three prime types equally frequently.

A total of 81 non-word targets were also selected for “NO” responses in a lexical decision task. The non-word targets consisted of three sets of 27 non-words which served as counterparts to the REG, IRLP, and IRLV verb targets. Within each set of non-word targets, a third of the targets (*n* = 9) were primed by words that mimicked the relationship between the morphological prime-target word pairs (e.g., father-FATH, slam-SLOG, ticket-TIVE). A third of the targets were primed by words that were orthographically similar to their targets (e.g., carbon-CARN, box-BOP, and nag-NAGE). A third of the targets were primed by words that were orthographically and phonologically unrelated (e.g., corner-TOAK, carry-PONER, and team-TATCH). As non-words do not have morphological representations, the implication is that within the 81 non-words, two-thirds of the targets were primed by orthographically (and also phonologically) similar word primes, and one-third by unrelated word primes. Lexical characteristics of the primes (e.g., mean word frequencies, lengths, numbers of neighbors, and orthographic similarity) were similar to their counterparts in the word target conditions. The lexical characteristics for the stimuli in the non-word target conditions are available in the Supplementary Material. Prime Type was not manipulated for non-words, and, therefore, there was only one presentation list for non-word targets. None of the word primes preceding non-word targets was used as a critical stimulus (i.e., in the word prime-target pairs).

#### Apparatus and Procedure

The presentations of the stimuli and the recording of responses were controlled by DMDX (Forster and Forster, 2003). Participants were tested individually in a quiet room. The presentation sequence of a trial was identical to that of Feldman et al. (2010) and was as follows: a fixation point (i.e., “+”) was first presented for 450 ms, which was followed by a 50 ms blank screen. Then, a string of number signs (i.e., “#”), matching the letter length of the prime, was presented for 500 ms as a forward mask. Immediately after the presentation of the forward mask, a prime was presented for 50 ms in lower-case letters, which was immediately replaced by a target in upper-case letters. Targets remained on the screen for 3,000 ms or until a response was made. The inter-trial interval was 1,000 ms. The stimuli were presented in 18 pt. Courier New font at the center of the display.

Participants were asked to decide whether each target stimulus is a real English word or not and indicate their decision by pressing the “YES” or “NO” button on a game pad (Tohoku University) or on a response box (Waseda University) as fast and accurately as possible. Prior to the presentation of the experimental trials, 36 practice trials were presented in order to familiarize participants with the task. Participants were asked to repeat the practice session until they felt comfortable with the task. The presentation lists were counterbalanced across participants and the order of trials within each list was randomized for each participant. Approval for the experiments was obtained from the ethics review board of the Graduate School of International Cultural Studies Tohoku University, and the ethics review committee on research with human subjects of Waseda University.

### Results

Data from two participants were removed because they made more than 25% errors (one participant each from List A and List C). To equate the numbers of participants between the presentation lists, data from one additional participant (the last participant from List B) were removed. As a result, data from 90 participants were analyzed. Responses with latencies greater than 1,500 ms were considered to be outliers (0.32% of word data) and were also removed from the entire analyses.

In the analysis of the response latencies, we analyzed the data with linear mixed effect (LME) models (e.g., Baayen et al., 2008) using the lme4 package (Version 1.1–27.1, Bates et al., 2015) available in R (Version 4.1.1, R Core Team, 2021). For the response latency analyses, a reciprocal inverse transformation was applied to the raw RTs (i.e., −1,000/RT; hereafter, *invRT*) to meet the assumption of normality. In order to calculate the *p*-values with the degrees of freedom based on Satterthwaite’s approximation, we used the anova function of the lmerTest package (Version 3.1–3, Kuznetsova et al., 2017). The model used was *invRT* ~ Verb Type*Prime Type + (1|Participants) + (1|Targets). In addition, *post-hoc* comparisons were carried out using the emmeans package (Version 1.7.2, Lenth, 2022) with Tukey’s HSD adjustments when necessary. The error analysis was conducted with the same procedure except that we used a generalized linear mixed-effect model, assuming a binomial distribution, and the anova function in the car package (Version 3.0–12, Fox and Weisberg, 2019) was used to obtain the *p* values for the fixed effects. The model used was Error ~ Verb Type*Prime Type + (1|Participants) + (1|Targets). Mean response latencies and error rates of Experiment 1 are shown in Table 2.

**Table 2:** Mean response latencies and (error rates) of targets primed by morphological, orthographic, and unrelated primes for Experiment 1.

Verb type	Prime type			Priming effect	
	MORPH (M)	ORTH (O)	UNREL (U)	O-M	UR-O
IRLP	606 (9.6)	612 (11.4)	647 (10.9)	6 (1.8)	35 (−0.5)
IRLV	596 (6.3)	600 (8.0)	622 (8.3)	4 (1.7)	22 (0.2)
REG	590 (8.5)	604 (7.2)	633 (8.2)	14 (−1.4)	29 (1.0)

IRLP = Irregular Length Preserved Verbs; IRLV = Irregular Length Varied Verbs; REG = Regular Verbs.

#### Response Latencies

The main effect of Prime Type was significant, *F*(2, 6458.8) = 91.29*, p <* 0.001. The main effect of Verb Type was not significant, *F < 1.* The interaction between Verb Type and Prime Type was not significant, *F*(4, 6458.9) = 1.22, *p* = 0.30, meaning that the patterns of priming effects were not different for REG, IRLP, and IRLV targets.

Follow-up analyses of the significant main effect of Prime Type revealed that across Verb Type, targets primed by morphological primes were responded to significantly faster than the same targets primed by unrelated primes, *estimated coef.* = −0.112, *SE* = 0.00861, *t* = −13.03, *p* < 0.001. Targets primed by orthographic primes were also responded to significantly faster than the same targets primed by unrelated primes, *estimated coef.* = −0.083, *SE* = 0.00863, *t* = −9.65, *p* < 0.001. Critically, targets primed by morphological primes were responded to significantly faster than targets primed by orthographic primes, *estimated coef.* = −0.029, *SE* = 0.00860, *t* = −3.36, *p* = 0.02.

#### Error Rates

No effects were significant, all *p*s > 0.25.

### Discussion

In Experiment 1, targets primed by orthographic primes were responded to significantly faster than targets primed by unrelated primes. This effect replicated the results of Nakayama and Lupker (2018), in which orthographically similar English word primes significantly facilitated lexical decision latencies to English targets for Japanese-English bilinguals and is also in line with what may be expected when encoding of orthographic form is fuzzy (e.g., time-TIDE < doll-TIDE). Although similar results have been found in L2 morphological priming experiments when the readers’ L1 was alphabetic (e.g., Diependaele et al., 2011), this result does contrast sharply with findings observed in orthographic neighbor priming experiments for L1 readers, where the direction of the effect is typically inhibitory (e.g., time-TIDE > doll-TIDE; Segui and Grainger, 1990; Davis and Lupker, 2006; Nakayama et al., 2008).

In Experiment 1, targets were also responded to significantly faster when they were primed by morphological primes than by unrelated primes. This facilitation from morphological primes can be orthographic, not necessarily morphological, in origin, because as was observed, orthographic similarity can facilitate bilinguals’ L2 lexical decision latencies. Nevertheless, in Experiment 1, the *post-hoc* analysis showed that across target verb types, the size of the priming effect was significantly larger from morphological primes than from orthographic primes, although the difference was numerically small (7 ms). This additional facilitation for morphological prime-target pairs over orthographic prime-target pairs seems to suggest that representations reflecting morphological relationships *do develop in the L2 English lexicons of Japanese-English bilinguals*. However, we need to point out that although there was no Prime Type by Verb Type interaction, suggesting that the priming advantage for morphological over orthographic primes was not different for REG, IRLP, and IRLV verbs, REG targets produced a larger numerical advantage (14 ms) than the other two verb types (6 ms and 4 ms, respectively). This pattern is a bit difficult to interpret because in the REG condition, prime-target orthographic similarity was higher for morphological primes (67%) than for orthographic primes (52%). Thus, in the REG condition, the priming effect from morphological primes could involve additional facilitation due to orthographic similarity, meaning that the present experiment might overestimate the size of the morphological priming effect in that condition.

On the other hand, prime-target orthographic similarity between morphological and orthographic primes was matched in the IRLP and IRLV conditions and, therefore, any priming advantage for morphological primes in those conditions, would make a strong case for the impact of morphology. When the data in the IRLP and IRLV conditions were analyzed (removing the data from the REG condition), however, the priming advantage for morphological over orthographic primes was not quite statistically significant, *F*(1, 2801.7) = 3.66*, p* = 0.056. Although the morphological priming advantage over orthographic priming was nevertheless significant when data in the REG condition alone were analyzed, *F*(1, 1375.2) = 9.35, *p* < 0.01, as noted above, this difference could partly be due to morphological primes having orthographic similarity. Thus, although there was an indication that morphological level representations in L2 English lexicons may develop for Japanese-English bilinguals, the evidence is not robust. Therefore, Experiment 2 was an effort to investigate this issue further.

## Experiment 2

One potential problem with the design of Experiment 1, which may have led to the somewhat ambiguous results, was that the number of the items selected was relatively small. In Experiment 1, for each of the three verb type conditions, 27 items were primed by three types of primes. Therefore, there were only 9 items per cell for any given participant. Although we attempted to address this problem by testing a large number of participants (*N* = 90), our results may, unfortunately, not have been as stable as we might have wished. Therefore, in Experiment 2, we selected a larger set of items as critical stimuli. In order to allow us to increase the item numbers, we dropped the IRLV condition. There are not many IRLV verbs that our bilinguals would be familiar with, and, thus, the inclusion of this condition in Experiment 1 made it difficult for us to have a large number of stimuli in the various conditions. Because we were not directly interested in investigating the effects of word length (varied or preserved) on the development of morphologically-based connections between past-tense verbs and their present-tense forms, removing the IRLV condition does not impede our research goal. Thus, in Experiment 2, only two verb type conditions were examined: regular verbs (REG; *n* = 48, e.g., look-LOOKED) and irregular verbs (IREG; *n* = 48, e.g., fell-FALL). We should acknowledge that, although equating orthographic similarity between morphological prime-target pairs and orthographic prime-target pairs was optimized, it was still not possible to fully equate the values for regular prime-target pairs given the limited vocabulary sizes of our bilinguals. To compensate, we conducted a *post-hoc* regression analysis to ascertain if greater orthographic overlap affected priming effects from morphological primes in the REG condition. In the IREG condition, orthographic similarity was fully matched between the morphological and orthographic prime-target pairs. Therefore, any priming effect observed relative to orthographic primes in this condition can be attributed to the prime and target’s morphological relationship.

### Method

#### Participants

Participants were 84 Japanese-English bilinguals recruited at Tohoku University (*n* = 44) and Waseda University (*n* = 40). They spoke Japanese as their first language and were reasonably proficient in English (they all had a TOEIC score of 605 or a TOEFL ITP score of 510 or higher).^4^ Thirty-eight of the participants were male and 46 were female. The age of participants (excluding one who did not report his/her age) at the time of the experiment was 21.65 (*SD* = 3.20). The age they started learning English was, on average, *M* = 9.74, (*SD* = 3.37). The time they had spent in an English-speaking region was, on average, 3.83 months (*SD* = 15.78). Participants each received a 1,000-yen gift card (roughly equivalent to US$9.00) for their participation.

#### Stimuli

The targets consisted of two types of verbs (Verb Type): irregular verbs (IREG) and regular verbs (REG). Irregular verbs (IREG, *n* = 48) were verbs which do not take the “-ed” ending in their past tense (i.e., their past tense is formed irregularly) and their past- and present-tense forms have the same letter length (e.g., fell-FALL). Regular verbs (REG, *n* = 48) were verbs which take the “-ed” ending to form the past tense (e.g., looked-LOOK). Each target was paired with three types of primes: morphological, orthographic, and unrelated primes. Examples and the lexical characteristics of the word targets are shown in Table 3.

**Table 3:** Lexical characteristics and examples of prime-target pairs used in Experiment 2.

		Prime type			Targets
		MORPH	ORTH	UNREL	
**IREG**		**fell**	**fill**	**joke**	**FALL**
	Frequency	105 (177.4)	120 (343.4)	97 (160.6)	371 (668.1)
	Length	4.2 (0.7)	4.2 (0.7)	4.2 (0.7)	4.2 (0.7)
	Neighbors	8.9 (5.3)	9.2 (5.6)	8.4 (4.5)	8.9 (4.5)
	% Overlap	64 (18.8)	66 (11.7)	0.0 (0.0)	
	LD	0.35 (0.17)	0.34 (0.12)	1.0 (0.00)	
**REG**		**looked**	**locker**	**rather**	**LOOK**
	Frequency	76 (112.2)	61 (234.3)	73 (102.3)	360 (545.7)
	Length	6.2 (0.6)	6.2 (0.6)	6.2 (0.6)	4.2 (0.6)
	Neighbors	5.3 (2.9)	2.2 (2.4)	2.2 (2.1)	9.3 (4.7)
	% Overlap	67 (2.8)	46 (10.2)	0.0 (0.0)	
	LD	0.49 (0.06)	0.78 (0.15)	1.47 (0.10)	

Values in word frequencies (per million words) and the number of neighbors were according to the English Lexicon Project (Balota et al., 2007). LD refers to the Levenshtein Distance (Levenshtein, 1966).

Targets in the IREG and REG conditions were matched on their mean word frequencies, word lengths, and numbers of neighbors (all *t*s < 1). For primes paired with IREG targets, the morphological, orthographic, and unrelated primes were matched on their mean word frequencies (*M* = 105, 119, 97, *F* < 1), word lengths (*M*s = all 4.2), and number of neighbors (*M*s = 8.9, 9.2, 8.4, *F* < 1). Prime-target orthographic overlap was statistically equivalent for morphological primes and orthographic primes (*M*s = 64 and 66%), *t* < 1. Unrelated primes had no orthographic overlap with their targets (*M* = 0%).

For the primes paired with REG targets, morphological, orthographic, and unrelated primes were matched in their mean word frequencies (*M*s = 76, 61, 73, *F* < 1) and word lengths (*M*s = all 6.2, as all primes for a given target had the same length). As was the case in Experiment 1, prime-target orthographic overlap was inevitably significantly higher for morphological primes (*M* = 67%) than for orthographic primes (*M* = 46%), *t*(47) = 13.03, *p* < 0.001, *SEM* = 1.62. Unrelated primes had no orthographic overlap with their targets (*M* = 0%). Morphological primes also had a statistically higher number of neighbors (*N* = 5.3) than orthographic (*N* = 2.2, *t*(47) = 6.92, *p* < 0.001, *SEM* = 0.46) or unrelated primes (*N* = 2.1, *t*(47) = 6.44, *p* < 0.001, *SEM* = 0.50), which did not differ from one another (*t* < 1).

A total of 96 non-word targets were also selected for “NO” responses. More than half of the non-words were generated with Wuggy (Keuleers and Brysbaert, 2010). The non-word targets consisted of two sets of 48 non-words which served as counterparts to the REG and the IREG verb targets. The non-word targets had similar mean word lengths and numbers of neighbors as those of the word targets. Non-words were paired with word primes in the same way as in Experiment 1. Lexical characteristics of the primes (e.g., mean word frequencies, lengths, numbers of neighbors, and orthographic similarity) were similar to those of their counterparts in the word target condition. The lexical characteristics for the non-word target condition are available in the Supplementary Material. None of the word primes preceding non-word targets were used in the critical stimuli (e.g., word prime-target pairs).

#### Apparatus and Procedure

The apparatus and procedure of Experiment 2 were identical to those in Experiment 1.

### Results

Data from six participants were removed due to high error rates (25% or more). Data from one additional participant were removed due to noncompliance with the instructions. Data for these participants were replaced by those from additional participants while maintaining the counterbalancing of the lists. Responses with latencies greater than 1,500 ms were considered as outliers and were removed from the analyses (0.69% of word data). Six items (4 REG verbs, “lick,” “jail,” “sail,” and “bust” and one IREG verb: “stink,” as well as one verb in the IREG condition which also has a regular ending: “lie” which has past-tense forms of both “lay” and “lied” depending on which meaning of “lie” is intended) were also excluded from the entire analyses because those words produced more than 40% error rates. The remaining response latencies and error rates were analyzed as in Experiment 1 with LME and GLM models, respectively, except that the categorical factor Verb Type now only had two levels (REG, IREG). Mean response latencies and error rates of Experiment 2 are shown in Table 4.

**Table 4:** Mean response latencies and (error rates) of targets primed by morphological, orthographic, and unrelated primes for Experiment 2.

Verb type	Prime type			Priming effect	
	MORPH (M)	ORTH (O)	UNREL (U)	O-M	UR-O
IREG	634 (9.2)	655 (11.9)	676 (12.1)	21 (2.7)	21 (0.2)
REG	627 (8.2)	656 (11.0)	675 (12.0)	29 (2.8)	19 (1.0)

IREG = Irregular verbs with the same character length between past and present forms; REG = Regular verbs.

#### Response Latencies

The main effect of Prime Type was significant, *F*(2, 6525.7) = 98.27, *p* < 0.001. The main effect of Verb Type was not significant, *F* < 1. The interaction between Verb Type and Prime Type was also not significant, *F*(2, 6525.8) = 1.35, *p* > 0.25. Therefore, as was the case in Experiment 1, patterns of priming effects were not significantly different for regular and irregular targets.

Follow-up analyses of the significant main effect of Prime Type revealed that across Verb Type, targets primed by morphological primes were responded to significantly faster than targets primed by unrelated primes, *estimated coef.* = −0.114, *SE* = 0.00815, *t* = −14.02, *p* < 0.001. Targets primed by orthographic primes were also responded to significantly faster than targets primed by unrelated primes, *estimated coef.* = −0.059, *SE* = 0.00821, *t* = −7.14, *p* < 0.001. Consistent with Experiment 1, targets primed by morphological primes were responded to significantly faster than targets primed by orthographic primes, *estimated coef.* = −0.056, *SE* = 0.00813, *t* = −6.84, *p* < 0.001. Although the interaction was not significant, we further analyzed the patterns of morphological priming effects separately for REG and IREG conditions. The results showed that in the IREG condition, targets primed by morphological primes were responded to 21 ms faster than targets primed by orthographic primes, and this advantage in processing speed was statistically significant, *F*(1, 2154.8) = 12.02, *p* < 0.001. In the REG condition, there was a significant 29 ms processing advantage for targets primed by morphological compared to orthographic primes, *F*(1, 2091.4) = 39.10, *p* < 0.001.

#### Error Rates

The only significant effect was the main effect of Prime Type, *X^2^* = 8.10, *p* = 0.017. As expected, across Verb Type, error rates were significantly smaller for targets primed by morphological primes (*M* = 8.7%) than by unrelated primes (*M* = 11.9%), *estimated coef. =* − 0.419*, SE* = 0.0985, *z* = −4.26, *p* < 0.001. Error rates were also significantly smaller for targets primed by morphologically related primes than targets primed by orthographic primes (*M* = 11.4%), *estimated coef. = −* 0.343*, SE* = 0.0993, *z* = −3.45, *p* < 0.01. Error rates were not statistically different for targets primed by orthographic primes versus unrelated primes, *z* < 1. These patterns did not interact with Verb Type, *X^2^* < 1, *p* > 0.75. Thus, paralleling the RT data, there was a significant morphological priming effect relative to both orthographic and unrelated primes. Separate analyses for the IREG and REG conditions also confirmed that morphological priming effects, measured against orthographic primes, were significant in both the IREG condition, *X^2^* = 5.99, *p* = 0.014, and the REG condition, *X^2^* = 6.08, *p* < 0.014.^5^

### Discussion

In Experiment 2, morphological primes facilitated lexical decisions to targets more than orthographic primes did. Importantly, this pattern did not appear to vary by verb type as indicated by the non-significant interaction. The results in Experiment 2, therefore, successfully replicated those in Experiment 1.

In the stimulus selection of Experiment 2, our priority was that our participants were reasonably familiar with English stimuli, especially the masked primes. As a result of this constraint, in the REG condition, morphological primes were more orthographically similar to their targets than the orthographic control primes were to their targets (67 and 46%, respectively). We were able to equate prime-target orthographic similarity between morphological and orthographic primes (64 and 66%, respectively) in the IREG condition, however. The difference in the orthographic overlap in the REG condition may be at least part of the reason why the morphological priming effect was larger than the orthographic priming effect in that condition. Thus, we decided to re-examine this issue. For individual targets, the size of the morphological priming effect (calculated from raw RTs) was regressed against the difference in the orthographic overlap between M-UR vs. O-UR pairs in the REG condition. Crucially, the size of the additional orthographic overlap had no association with the size of morphological priming effect observed in the REG condition (*t* < 1, *β* = 0.12, *n.s.*). Together with the fact that Verb Type did not significantly interact with the pattern of morphological priming effects, it seems safe to conclude that the morphological priming effect observed in the REG condition, when measured against orthographic primes, reflected mainly the impact of the primes’ and targets’ morphological similarity.

## General Discussion

In the present research, we investigated whether representations of past-tense verbs and their present-tense forms in the lexicons of L2 readers are organized in the same way as is assumed to be the case in the lexicons of English L1 readers. The results of the two experiments suggest that connections based on the morphological relationships of past- and present-tense verbs in the L2 English lexicon are similar to those of L1 English readers, at least in the population of Japanese-English bilinguals examined in the present research. Although the sensitivity of L2 readers to the morphological structure of complex words might be prone to fuzziness, our data seem to indicate that the morphological representations in our participants’ lexicons were not organized in an imprecise manner (at least with respect to the representations of past-tense verbs and their present-tense forms). In Experiment 1, past-tense verb primes facilitated lexical decisions to their present-tense targets compared to both an orthographic and an unrelated baseline. The results of Experiment 1 were, however, not entirely clear because it was difficult to dissociate the impact of prime-target orthographic relationships from those of morphological relationships. Nevertheless, the results of Experiment 2, which had a better controlled and larger set of stimuli, replicated the general data pattern of Experiment 1. That is, past-tense primes facilitated responses to their present-tense targets when compared to an orthographic baseline and such was the case for both regular and irregular verbs. Therefore, the way English past-tense verbs and their present-tense forms are connected in the lexicons of Japanese-English bilinguals appears to be reasonably similar to the way they are connected in the lexicons of L1 English readers. Fuzziness in encoding L2 word forms, manifesting itself as facilitory priming effects from orthographically related primes, did not lead to differences in the way that past-tense verbs and their present-tense forms were connected.

We should note, however, that the exact nature of such connections in L1 readers, especially when considering the representations of regular versus irregular verbs, is not yet agreed upon. Some researchers believe that the past- and present-tense forms of regular verbs and irregular verbs are connected in qualitatively different ways. One common view is that regular verbs share the same underlying representation through the common root (i.e., boiled - boil *via* the shared root “boil”), while irregular verbs have separate underlying representations with tight connections *via* semantic and formal association (i.e., “fell” and “fall” have separate and independent representations). Other researchers believe that there is no qualitative difference in how regular and irregular verbs are represented. One variant of the latter view further assumes that both types of past-tense and present-tense verbs are connected *via* their semantic and formal relationships with some models not assuming the presence of explicit morphological level representations. Another variant assumes that both types of past-tense and present-tense verbs have shared representations *via* the morphological root or lemma. Discussion of exactly how past-tense and present-tense verbs are connected in English L1 lexicons is beyond the scope of the present research (please see Feldman and Weber, 2012; Milin et al., 2018, for reviews). However, the main point here is that, whatever account of L1 connections is accurate, the similar patterns of morphological priming observed for regular and irregular verb targets in our experiments provide no evidence in support of a different morpho-syntactic process for L2 readers than for L1 readers (*cf.*, Silva and Clahsen, 2008; Clahsen et al., 2013). In that sense, our results are in line with the view that seems to be more widely accepted in the current L1 literature, that the two types of past-tense and present-tense verbs are represented similarly, with their word forms being connected in a way that goes beyond just a sum of form and semantic similarity (e.g., Stockall and Marantz, 2006; Kielar et al., 2008; Crepaldi et al., 2010; Morris and Stockall, 2012; Fruchter et al., 2013).

### Effects of L2 Proficiency on Morphological and Orthographic Priming Effects

According to the fuzzy lexicon hypothesis, L2 lexical representations do not inevitably remain fuzzy but rather become more robust with more experience with the L2 input (which would lead to a higher lexical quality of words). In Experiment 2, we recruited a large number of participants who encountered a larger set of stimuli per condition than in Experiment 1. To gain some additional understanding of the development of the L2 lexicon, we examined how L2 proficiency affected the patterns of morphological priming effects for our Japanese-English participants. In these *post-hoc* analyses of L2 proficiency, TOEIC scores were used. The analyses were based on 66 participants who had their TOEIC scores available (79% of the participants in Experiment 2). Their mean TOEIC score was 790.76 (*SD* = 93.33, range: 605–965).

**Figure 1:**
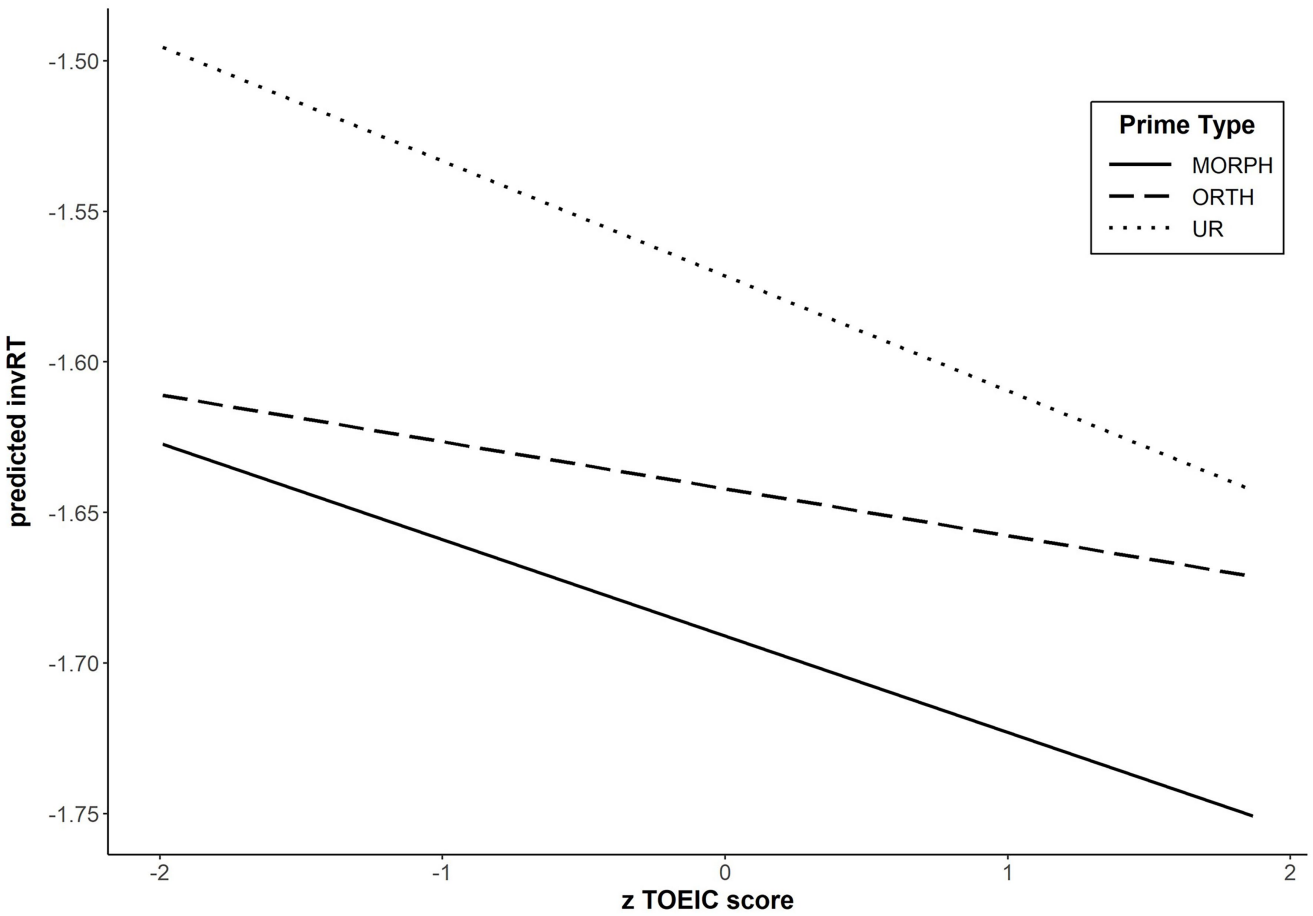
Response latencies to L2 targets primed by morphological, orthographic, and unrelated primes and priming effects as a function of L2 proficiency in Experiment 2. Greater zTOEIC score indicates higher proficiency, and smaller invRT indicates faster responses to L2 targets.

In the L2 proficiency analyses, TOEIC scores were first converted to z scores and entered as a factor. The model used was *invRT* ~ Verb Type*Prime Type*zTOEIC score + (1|subject) + (1|target). The results showed no main effect of L2 Proficiency (i.e., zTOEIC score), *F*(1, 64.1) = 1.11, *p* = 0.30, indicating that overall responding speed to targets was not modulated by TOEIC scores. L2 proficiency, however, significantly interacted with Prime Type, *F*(2, 5152.0) = 3.02, *p* = 0.049. L2 Proficiency did not interact with Verb Type, *F* < 1. The three-way interaction between Proficiency, Verb Type, and Prime type was also not significant, *F* < 1. Figure 1 shows the pattern of the two-way interaction observed in the L2 proficiency analyses.^6^

Because the main issue here is the impact of L2 proficiency on morphological priming, we first investigated the difference between the morphological and orthographic priming conditions. L2 proficiency did not significantly interact with the pattern of morphological priming, *F*(1, 3456.3) = 1.64, *p* = 0.20. However, as can be seen in Figure 1, L2 proficiency appears to be affecting morphological priming effects quite differently at the two extreme levels of proficiency, with the effect being considerably smaller for less proficient bilinguals. To further assess the patterns of morphological priming effects, we statistically tested the priming effects for the most and the least proficient bilinguals, using the *emmeans* package (Version 1.7.2, Lenth, 2022). For participants with the highest L2 proficiency (*z* = 1.87; the raw TOEIC score = 965), the morphological priming effect defined this way was significant*, estimated coef.* = −0.072, *SE =* 0.0207, *t =* −3.46*, p* < 0.001. For participants with the lowest L2 proficiency (*z* = −2.01; the raw TOEIC score = 605), however, there was no clear sign of a morphological priming effect, *t =* −1.05*, p* = 0.30.

Next, we considered the impact of L2 proficiency on the orthographic priming effects (i.e., the difference between the orthographic and unrelated conditions). L2 proficiency significantly affected this priming pattern, *F*(1, 3376.9) = 5.78, *p* = 0.016. The modulation emerged because orthographic priming became smaller as L2 proficiency increased. When the priming patterns for the most and least proficient bilinguals were separately analyzed, the most proficient bilinguals did not show a significant orthographic priming effect, *estimated coef.* = −0.026, *SE =* 0.0208, *t =* −1.27*, p* = 0.20. For the least proficient bilinguals, on the other hand, there was a significant orthographic priming effect, *estimated coef.* = −0.118, *SE =* 0.0218, *t =* −5.41*, p* < 0.001.

With regard to the pattern of orthographic priming effects, the observed interaction is a result that was different from that of Nakayama and Lupker (2018) who found no modulation by L2 proficiency (TOEIC score) on the magnitude of orthographic priming effects. We are not entirely sure why L2 proficiency differently affected the patterns of orthographic priming effects here. We could, however, speculate that the presence of morphologically related pairs, which were not presented in Nakayama and Lupker (2018), may have somehow affected how high proficient bilinguals deal with orthographic similarity. Although inconsistent with the results of Nakayama and Lupker, numerically, our data pattern does converge with Feldman et al.’s (2010) Serbian-English bilinguals’ data in that facilitation from orthographic primes was smaller for their more proficient bilinguals (12 ms) than for their less proficient bilinguals (26 ms). This pattern could be accounted for by assuming that there is a high degree of fuzziness in the low-proficient bilinguals’ orthographic lexicon which might then lead to weaker competition between lexical items in this group (thereby increasing facilitation from orthographically similar word primes).

Finally, L2 proficiency did not significantly modulate morphological priming effects relative to unrelated primes, *F*(1, 3444.8) = 1.23, *p* = 0.27. As shown in Figure 1, there was no significant difference in the sizes of the morphological priming effect between the most and least proficient bilinguals. Given the fact that the orthographic priming effect was significant only for the least proficient bilinguals, the apparent morphological priming effect for less proficient bilinguals seems to be driven mostly by the prime-target orthographic similarity.

As such, the overall picture is that, although L2 proficiency did not modulate the patterns of morphological priming effects when measured using an unrelated baseline, L2 proficiency affected the pattern of orthographic priming effects and, hence, the degree to which orthographic priming effects contribute to the overall morphological priming effects. A reasonable account of this difference is that for those with a higher level of L2 visual word recognition skills, lexical competition starts to operate in the recognition of L2 words (orthographic representations become less fuzzy). Therefore, orthographic similarity no longer is an effective source of facilitation from word primes. The *post-hoc* analyses suggest that weak lexical competition, a characteristic of the L2 lexicon, will likely strengthen with greater L2 exposure, at least when an experimental input is visual (as opposed to, for example, the auditory domain, in which representations of some difficult-to-distinguish phoneme contrasts may not inevitably become of high quality due to greater amount of exposure). In contrast, with greater proficiency, the morphological relationships become more stable in the L2 lexicon. As a result, priming based on those relationships rather than orthographic relationships becomes more potent.

### Orthographic and Morphological Level Representation in the L2 Lexicon

In the early stages of visual word recognition, activation in the lower-level representations is assumed to flow to upper-level representations *via* feed-forward connections. An obvious implication of this assumption is that the correct activation of a higher-level representation must, in some way, depend on the correct activation of lower-level representations. Lexical competition is one mechanism that would, presumably, prevent the lexical system from erroneously activating semantic and other upper-level representations of words not being presented (by suppressing any competitors at the lexical level). Hence, lexical competition would seem to be a mechanism that would need to be developed somewhat early by L2 readers. Nonetheless, L2 readers appear to have weak lexical competition operations (Nakayama and Lupker, 2018). Therefore, for those individuals, co-activation of orthographically related words is likely to occur frequently, leading to incorrect activation of the upper-level representations (e.g., lexical/semantic representations of orthographically similar words to the target would be co-activated). When there are only weak lexical competition operations, as seems to be the case for many L2 readers, it seems that it would be difficult for upper-level representations to become firmly established unlike in the L1 lexicon. Indeed, in the present experiments, our results suggest something of this sort is occurring. That is, when L2 lexical representations are weak (i.e., for the participants with lower TOEIC scores and larger orthographic priming effects), it is difficult for morphological representations to produce priming. However, as L2 readers become more skilled, their orthographic representations become less fuzzy, their orthographic priming effects become smaller, and priming based on morphological relationships alone becomes larger.

All this is not to say, however, that lower-level representations need to be fully developed before higher-level representations start to develop. Recently, Bordag et al. (2021) proposed the Ontogenesis Model (OM) of L2 lexical representations, which may provide further insight with respect to this issue. The OM is a model that provides a blueprint of the nature of L2 lexical representations and their development at the differential phases of L2 attainment. The OM posits that a central characteristic of the L2 lexicon is fuzziness in how words are represented and connected, and a weak form-based competition is one result of this fuzziness in L2 orthographic representations. Importantly, the OM also has the ability to explain how the development of higher-level representations is not as strongly affected by the development of lower-level representations in the L2 lexicon as one might assume. Of particular relevance, the OM acknowledges that L2 lexical acquisition is different from L1 lexical acquisition in that, although lexical acquisition in L1 involves the simultaneous development of both form and semantic representations, “*lexical acquisition in L2 often involves the establishment of a new form representation and its mapping onto an existing semantic representation*” (p: 10). The connection between form-level representations and upper-level (semantic) representations is therefore somewhat weaker in L2. The OM also posits that the weaker form to meaning link in L2 makes it difficult for co-activated (i.e., incorrect) orthographic representations to activate their upper-level representations. However, a link between a given L2 word form and its meaning is typically established differently than would be the case in L1. That is, that link is established as a result of mapping the existing semantic knowledge to the word form. Under this view, co-activation of orthographically similar words at the orthographic level does not substantially hinder the correct semantic activation of a target word. The OM in its current form does not yet incorporate representations involving morphological relationships (see Footnote 4, Bordag et al., 2021). However, it seems possible to apply the model’s principles to the mappings between L2 forms and morphological/lemma level representations. That is, the present-tense and past-tense morphological relationship in the L1 language, which has been established at the conceptual level (e.g., aruku-aruita, utau-utatta; Japanese), can be directly mapped onto present- and past-tense forms in L2 (e.g., walk-walked, sing-sang; English).

### The Contrast Between Experiment 2 and the Previous Studies Testing Past-Tense Morphological Priming in L2

In the present experiments, we largely followed the design of Feldman et al. (2010) by employing both an orthographic and an unrelated baseline. In terms of priming effects relative to unrelated primes, our results were largely consistent with what Feldman et al. observed—priming effects were significant, and they did not interact with verb type. However, our results were different in terms of priming effects relative to orthographic primes (particularly in Experiment 2); while we observed significant morphological priming effects for both regular and irregular verbs, Feldman et al. observed significant effects for regular verbs only.

One possible reason for the discrepancy is that there may have been insufficient power in Feldman et al.’s (2010) design. Although the number of participants in Feldman et al.’s experiment was large (*N* = 90), the number of items for each verb type was small (*n* = 21), which meant that, with three prime types for each target verb type, there were only seven items per cell. Further, numerically, their morphological priming effects when measured against orthographic primes were non-zero (2-19 ms). For irregular verbs, for example, the priming effect was as large as 14 ms. Our Experiment 1, using a similar number of participants and number of items as used by Feldman et al., also suffered from some ambiguity in its data pattern as it produced only small priming effects that were not significant when considered individually. It is possible that Feldman et al.’s experiment, as well as the present Experiment 1, did not have enough power to detect small differences.

Another possible explanation for the observed difference between the results in our Experiment 2 and Feldman et al.’s (2010) is that there was a difference in the word frequencies of targets used in the two experiments. In the present experiments, we selected our stimuli in such a way that our bilinguals would be quite familiar with the words (both primes and targets). As a result, our targets were very high frequency words (>350 occurrences per million), which we assumed that bilinguals would have had abundant experience with. On the other hand, target words in Feldman et al.’s experiments were of medium frequency (60–85 occurrences per million). A reflection of this difference can be seen in the speed of overall responses. In Feldman et al.’s experiments, the bilinguals’ mean RTs ranged from 725 to 800 ms. In the present experiments, they ranged from 591 to 676 ms. In fact, the RT ranges observed for our Japanese-English bilinguals were quite similar to those observed for the L1 English speakers in Feldman et al.’s experiments (606–664 ms). Hence, it is likely that the representations of words used in our experiments had a higher overall level of entrenchment for our bilinguals. If L1-like connections do become established in the L2 lexicon with experience, such connections would be more likely to exist for words bilinguals encounter and process more often. The discrepancy between their experiments and ours may be that our high-frequency English stimuli allowed us to look at the connections that had become more entrenched than the connections that Feldman et al.’s stimuli allowed them to examine. In effect, word familiarity would be working in the same way as participant proficiency (see Figure 1).

As noted, in terms of priming effects from morphological primes relative to unrelated primes, our results were consistent with Feldman et al. (2010): both regular and irregular past-tense forms primed their present-tense targets. This result, however, is inconsistent with the findings for L2 readers reported by Silva and Clahsen (2008) and Clahsen et al. (2013), who found no priming for regular past-tense forms relative to unrelated primes. Although we do not know why Silva and Clahsen and Clahsen et al. did not observe priming for L2 readers, some possible sources of the discrepancy may include variables relating to list context such as the number of filler trials and type of non-word targets in the stimuli, as well as the frequency of word items in the experiment.

Participant variables such as the participants’ first language background and L2 proficiency could also be at play with respect to this discrepancy. First, we consider the possibility of proficiency differences. Our results with Japanese-English bilinguals suggest that the morphological condition should facilitate target responses relative to unrelated controls for both more proficient and less proficient bilinguals, albeit for slightly different reasons. The facilitation is likely to be mainly driven by prime-target orthographic overlap for less proficient participants, whereas it is likely more due to the prime-target morphological relationship for proficient participants. Therefore, a lack of priming based on morphological relationships could be explained if the participants of Silva and Clahsen (2008) and Clahsen et al. (2013) were overall less proficient than ours. Unfortunately, we cannot compare the proficiency measure used in our *post-hoc* analysis (i.e., the TOEIC) to Silva and Clahsen and Clahsen et al.’s analysis (i.e., the Oxford Placement Test).

What is also puzzling is why the regular past-tense primes in Silva and Clahsen (2008) and Clahsen et al. (2013) did not facilitate recognition of present-tense targets based on orthographic similarity, because the orthographic overlap between prime-target pairs has consistently been observed to facilitate target recognition for L2 readers (e.g., the present experiments; Nakayama and Lupker, 2018; Jiang, 2021; Kida et al., 2022). One possibility is that the nature of the bilinguals’ L1 versus L2 script could have affected lexical competition, as discussed by Nakayama and Lupker (2018): lexical competition operates more readily for same-script bilinguals than different-script bilinguals. Thus, in the case of same-script bilinguals, the orthographic similarity of L2 words would inhibit (or facilitate more weakly) target recognition. If those same-script bilinguals are also less proficient in the L2 and do not (yet) possess sufficient morphological processing skills, morphological primes will not facilitate target recognition relative to unrelated primes.

## Conclusion

Previous studies have shown that connections between past-tense verbs and their present-tense forms exist in native (L1) English readers’ lexicons. Our aim was to examine whether such connections are established in L2 English readers’ lexicons. The results of two masked priming lexical decision experiments with Japanese-English bilinguals showed that morphologically related primes facilitated lexical decisions to targets more than orthographically related primes. This pattern suggests that facilitation occurs due to the primes’ morphological relationship with their targets. Hence, our results demonstrate that connections in terms of past-tense morphology develop for L2 readers for both regular and irregular verbs. This result also indicates that at the morphological level, there is little imprecise (or fuzzy) encoding with respect to representations of morphological relationships. The present experiments also demonstrated that orthographic similarity of word primes and their targets facilitates target recognition for L2 readers, and thus underlines the importance of using orthographic primes in order to correctly gauge the impact of the underlying connections based on morphological relationships.

We would, therefore, like to advocate that subsequent studies should continue to use orthographic, in addition to, unrelated primes when attempting to gauge the impact of morphological priming effects in L2. With regard to the effects of orthographic similarity for L2 readers, significant facilitation effects have been observed with different-script bilinguals (Li et al., 2017a,b; Nakayama and Lupker, 2018; Jiang, 2021). Interestingly, the current models of bilingual visual word recognition (e.g., Bilingual Interactive Activation Model, Dijkstra et al., 1998; Bilingual Interactive Activation Model+, Dijkstra and van Heuven, 2002) do not assume that orthographically similar primes produce facilitation effects for same-script bilinguals (e.g., French-English bilinguals, Dutch-English bilinguals). Empirically, however, orthographic primes do appear to facilitate L2 lexical decision latencies for same-script L2 readers based on data from morphological priming experiments (e.g., Diependaele et al., 2011; Heyer and Clahsen, 2015). Therefore, the models cannot be precisely correct. The notion of form-prominence resulting from fuzziness in L2 encoding of orthographic form, however, would offer an explanation for these empirical findings (See Footnote 1). The presence and degree of orthographic facilitation appears to be affected by a number of factors, such as L1 orthography, L2 proficiency, stimulus compositions, etc. Thus, until we have a clearer understanding of orthographic priming effects on L2 visual word recognition, it would be advisable to use an orthographic baseline in addition to an unrelated baseline in order to make sure that the effects of orthographic (form) and morphological relationships are fully dissociated.

## Data Availability Statement

Readers interested in the data supporting the conclusions of this article may contact the corresponding author.

## Ethics Statement

The studies involving human participants were reviewed and approved by the Ethics Review Committee on Research with Human Subjects, Waseda University and the Ethical Review Board of the Graduate School of International Cultural Studies, Tohoku University. The participants provided their written informed consent to participate in these studies.

## Author Contributions

MN contributed to the conception and design of the study. JW-K and MN prepared the experimental stimuli. JW-K and MY collected data and prepared it for analysis. MN, MY, and JW-K performed statistical analysis. JW-K wrote the initial draft of the manuscript. MN, MY, SL, RV, and JW-K wrote sections of the manuscript. All authors contributed to the article and approved the submitted version.

## Funding

This work was supported by JSPS KAKENHI Grant Number JP19K14468.

## Conflict of Interest

The authors declare that the research was conducted in the absence of any commercial or financial relationships that could be construed as a potential conflict of interest.

## Publisher’s Note

All claims expressed in this article are solely those of the authors and do not necessarily represent those of their affiliated organizations, or those of the publisher, the editors and the reviewers. Any product that may be evaluated in this article, or claim that may be made by its manufacturer, is not guaranteed or endorsed by the publisher.

## Footnotes

^1^Gor, K., Cook, S., Bordag, D., Chrabaszcz, A., and Opitz, A. (under review; this issue). Fuzzy lexical representations in adult second language speakers. Front. Psychol. 12.

^2^Some previous studies with L1 readers have subdivided irregular verbs into those that are more irregular and less irregular, with results suggesting that inflectional regularity may have gradable effects on the processing of past-tense forms (e.g., Kielar et al., 2008; Kielar and Joanisse, 2010). We did not take this approach, because it requires the use of a much larger number of verbs (with greater variability in their orthographic similarity). We stuck to 2–3 categorization of verbs because we wanted to ensure that L2 readers are well familiar with the past-tense forms of irregular verbs, and this restriction forced us to select small sets of irregular verbs. There presentations of past-test verbs being robust in L2 readers’ lexicons is critical because if they are not robust, morphological facilitation would be confounded with orthographic facilitation.

^3^The TOEIC and TOEFL are developed and administered by the Educational Testing Service (ETS) and assess the English abilities of non-native English speakers. The TOEIC test includes listening and reading comprehension questions with content related to daily communication and business. Its test scores range from 10 to 990. The TOEFL ITP is designed for administration at institutions, such as universities. It also includes listening and reading comprehension questions. The TOEFL test focuses on English used in academic settings, and its test scores range from 310 to 677. The EIKEN is a test for English communication administered by the Eiken Foundation of Japan and backed by Japan’s Ministry of Education, Culture, Sports, Science, and Technology. It tests general English reading, writing, listening, and speaking skills. It has seven levels, with Grade 1 indicating the highest English proficiency. EIKEN, unlike TOEIC and TOEFL, adapts a pass/fail system. An EIKEN Grade 2 certificate holder is assumed to have the English proficiency level of a high-school graduate (6 years of English learning in an academic setting) or higher. In Experiment 1, of the 90 participants whose data were analyzed, 68 had taken the TOEIC, and their mean score was 798.43 (*SD* = 102.89, range: 485–970). Thirty-five participants had taken the TOEFL ITP, and their mean score was 554.74 (*SD* = 25.33, range: 520–647). Forty-five participants had taken the EIKEN with grades ranging from grade 3 to grade 1. Many participants had scores for multiple English proficiency tests. Individuals were invited to participate in the experiments as long as they satisfied one of the test score criteria.

^4^Of the 84 participants whose data were analyzed, 66 had taken the TOEIC. Their TOEIC mean score was 790.76 (*SD* = 93.33, range: 605–965). Thirty-six participants had taken the TOEFL ITP. Their mean score was 552.00 (*SD* = 35.60, range: 485–650).

^5^We removed the random intercept for subjects in the error analyses as the model did not converge when it was included.

^6^We also analyzed the effects of the age of acquisition (AoA) and the time they had spent in an English-speaking region (length of residence; LOR) using the LME models. For AoA, there was a significant main effect of AoA, with faster AoA leading to faster responses to targets, *F*(1, 82) = 4.04, *p* = 0.048. However, importantly, AoA did not interact with any of the priming effects, *F*(2, 6,522,1) = 1.73, *p* = 0.18. The three-way interaction between AoA, Prime Type, and Verb Type was also not significant, *F*(2, 6522.2) = 1.04, *p* = 0.35. For LOR, no effects relating to priming effects were significant, all *F*s < 1.
