# Supplementary material for: Morphological Priming Effects in L2 English Verbs for Japanese-English Bilinguals
Source: Front Psychol. 2022 Jul 28;13:742965. doi: 10.3389/fpsyg.2022.742965 (PMC9366885; doi:10.3389/fpsyg.2022.742965)
Supplement: Supplementary file 1 [file Data_Sheet_1.docx]

Supplemental Materials

*Lexical Characteristics and Examples of Prime Nonword Targets Pairs used in Experiment 1*

|  | | Prime type | | |
| --- | --- | --- | --- | --- |
|  | | MORPH | ORTH | UNREL |
| **REG** |  | **father-FATH** | **carbon-CARN** | **corner-TOAK** |
|  | Frequency | 101 (175.6) | 26 (47.8) | 76 (104.2) |
|  | Length | 6.1 (0.3) | 6.1 (0.6) | 6.1 (0.3) |
|  | Neighbors | 4.1 (1.5) | 1.8 (3.0) | 3.4 (2.7) |
|  | % Overlap | 65.6 (3.0) | 50.2 (9.9) | 7.4 (8.3) |
| **IRLP** |  | **slam-SLOG** | **box-BOP** | **carry-PONER** |
|  | Frequency | 65 (53.7) | 49 (64.4) | 113 (166.8) |
|  | Length | 4.1 (0.3) | 4.2 (0.6) | 4.4 (0.7) |
|  | Neighbors | 8.6 (4.2) | 9.0 (6.3) | 6.0 (5.1) |
|  | % Overlap | 67.8 (10.6) | 53.5 (11.0) | 4.4 (8.3) |
| **IRLV** |  | **ticket-TIVE** | **nag-NAGE** | **team-TATCH** |
|  | Frequency | 82 (114.1) | 69 (57.9) | 55 (49.2) |
|  | Length | 4.6 (1.1) | 4.3 (1.2) | 4.3 (1.2) |
|  | Neighbors | 7.1 (5.2) | 7.4 (4.6) | 7.0 (4.5) |
|  | % Overlap | 56.7 (21.6) | 56.9 (24.0) | 11.1 (17.1) |

Note: Values in word frequencies (per million words) and the number of neighbors were according to the English Lexicon Project (Balota et al., 2007).

*Lexical Characteristics and Examples of Prime Nonword Targets Pairs used in Experiment 2*

|  | | Prime type | | |
| --- | --- | --- | --- | --- |
|  | | MORPH | ORTH | UNREL |
| **IREG** |  | **scarf-SCALM** | **mild-MIRD** | **snare-BRINT** |
|  | Frequency | 92 (124.0) | 68 (81.4) | 107 (177.5) |
|  | Length | 4.3 (0.8) | 4.2 (0.5) | 4.3 (0.6) |
|  | Neighbors | 8.3 (5.3) | 9.4 (5.3) | 8.1 (5.3) |
|  | % Overlap | 67.5 (9.8) | 68.5 (10.5) | 0.0 (0.0) |
| **REG** |  | **rumble-RUMB** | **shield-SAZE** | **chaser-LARE** |
|  | Frequency | 42 (133.0) | 65 (188.9) | 92 (146.7) |
|  | Length | 6.1 (0.5) | 6.1 (0.7) | 6.1 (0.5) |
|  | Neighbors | 4.9 (2.1) | 2.3 (3.0) | 2.1 (1.9) |
|  | % Overlap | 67.1 (2.6) | 43.3 (14.1) | 0.0 (0.0) |

Note: Values in word frequencies (per million words) and the number of neighbors were according to the English Lexicon Project (Balota et al., 2007).
